# DNA-Based Faecal Dietary Analysis: A Comparison of qPCR and High Throughput Sequencing Approaches

**DOI:** 10.1371/journal.pone.0025776

**Published:** 2011-10-06

**Authors:** Dáithí C. Murray, Michael Bunce, Belinda L. Cannell, Rebecca Oliver, Jayne Houston, Nicole E. White, Roberto A. Barrero, Matthew I. Bellgard, James Haile

**Affiliations:** 1 Australian Wildlife Forensic Services and Ancient DNA Laboratory, School of Biological Sciences and Biotechnology, Murdoch University, Murdoch, Western Australia, Australia; 2 School of Biological Sciences and Biotechnology, Murdoch University, Murdoch, Western Australia, Australia; 3 Centre for Comparative Genomics, Murdoch University, Murdoch, Western Australia, Australia; Institut de Biologia Evolutiva - Universitat Pompeu Fabra, Spain

## Abstract

The genetic analysis of faecal material represents a relatively non-invasive way to study animal diet and has been widely adopted in ecological research. Due to the heterogeneous nature of faecal material the primary obstacle, common to all genetic approaches, is a means to dissect the constituent DNA sequences. Traditionally, bacterial cloning of PCR amplified products was employed; less common has been the use of species-specific quantitative PCR (qPCR) assays. Currently, with the advent of High-Throughput Sequencing (HTS) technologies and indexed primers it has become possible to conduct genetic audits of faecal material to a much greater depth than previously possible. To date, no studies have systematically compared the estimates obtained by HTS with that of qPCR. What are the relative strengths and weaknesses of each technique and how quantitative are deep-sequencing approaches that employ universal primers? Using the locally threatened Little Penguin (*Eudyptula minor*) as a model organism, it is shown here that both qPCR and HTS techniques are highly correlated and produce strikingly similar quantitative estimates of fish DNA in faecal material, with no statistical difference. By designing four species-specific fish qPCR assays and comparing the data to the same four fish in the HTS data it was possible to directly compare the strengths and weaknesses of both techniques. To obtain reproducible quantitative data one of the key, and often overlooked, steps common to both approaches is ensuring that efficient DNA isolation methods are employed and that extracts are free of inhibitors. Taken together, the methodology chosen for long-term faecal monitoring programs is largely dependent on the complexity of the prey species present and the level of accuracy that is desired. Importantly, these methods should not be thought of as mutually exclusive, as the use of both HTS and qPCR in tandem will generate datasets with the highest fidelity.

## Introduction

DNA-based dietary analysis of faecal material has emerged as a promising tool to study animal biology, ecology and archaeology [1]–[4]. Dietary analysis is not limited to the discovery of what an animal consumes; it can also give an insight into ecosystem health [5]–[7], species' responses to environmental/anthropogenic stresses [8], and assist in the development of targeted strategies for conservation [9]. It is evident from the increase in the use of genetic techniques that there is a growing appreciation of the use of DNA-based faecal methods to investigate diet. The analysis of faecal material has proven to be a welcome move away from more invasive techniques used to study animal diet such as lethal sampling [10] and stomach flushing [11], both of which have undesirable effects on the sampled population [12]. Moreover, a general move towards molecular based approaches, e.g. fatty acid, stable isotope or DNA analysis, has allowed a shift from more subjective morphological approaches [1], [13]. The extraction and sequencing of DNA from faecal samples is seen to be an effective and reliable indicator of species' diet, offering increased specificity and taxonomic resolution compared to other techniques [14]–[16]. The possibility of misidentification of species is greatly reduced [14], [17] and the ability to account for a wider range of species within the actual diet is greatly increased when compared to morphology which relies entirely on analysis of undigested remains, therefore neglecting prey that may leave little trace of its consumption [18]–[20].

DNA based quantitative estimates of diet, however, are not without problems. Issues have arisen as a result of primer biases and the problem of differential digestion still remains. Put simply, “is what goes in what comes out” [21]? Moreover, variability in the amount of DNA per unit biomass between species and different tissues is also difficult to quantify. Attempts to address such concerns have recently become an active area of research. Such efforts include; the use of blocking primers to circumvent the issue of predator DNA amplification [7], [22]; the use of captive feeding trials to examine differential digestion; [21] and the introduction of correction factors to account for DNA amount variability within species and tissues [23]. These confounding factors continue to be a contentious issue within analytical dietary research, however, DNA-based methods arguably still present the best way forward in the explication of species' diet [1], [19].

**Figure 1:**
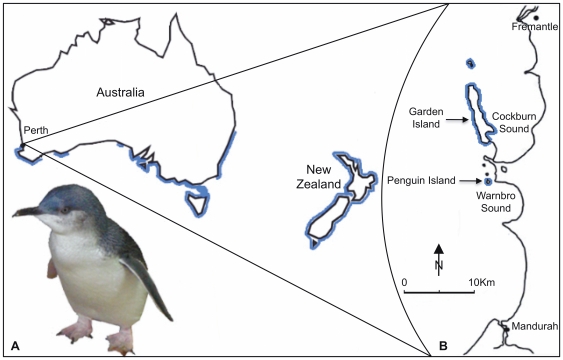
*Eudyptula minor* distribution and study site for faecal monitoring. (**A**) The costal distribution (marked in blue) of *E. minor* across Australia and New Zealand. (**B**) Map of the study site in Western Australia; for this faecal monitoring study samples were collected from Penguin Island.

Little Penguins (*Eudyptula minor*) are ideal test subjects for molecular dietary analysis and have been the subject of previous research into diet [21], [24]–[27]. The use of seabirds as barometers of marine ecosystem health is widely acknowledged, and the use of facultative feeders such as Little Penguins, whose diet is limited by food availability, provides a good indication of changes in marine environments [28], [29]. Little Penguins are found across the coastal regions of Australia and New Zealand [30] (Fig. 1) and their diet, which includes a variety of small (<20cm) schooling fish, varies throughout the year [24]–[27]. The penguin population situated on Penguin and Garden Islands (32°S 115′E), located south of Perth, Western Australia, represent the northernmost and westernmost limits of the range of *E. minor* [31], [32] (Fig. 1). As a fringe population, these penguins are more vulnerable to environmental changes such as rising sea temperatures and increased ocean acidification [33], [34]. Moreover, Penguin Island's close proximity to human settlement also puts it under increased pressure due to anthropogenic stressors, such as commercial and recreational fishing, in addition to coastal development [31], [35]–[38]. The development of a multi-year DNA-based study to investigate dietary preferences will prove an effective method to monitor *E. minor* and the marine environment.

Three major DNA-based techniques have been used to varying degrees in the study of species' diet. Firstly, PCR amplification using universal primers with subsequent cloning and sequencing of amplicons, is a technique that has been used extensively in molecular dietary analyses, and to some extent still is [13], [14], [39]. Secondly, quantitative PCR (qPCR), using species-specific primers has been purported to offer great promise in relation to dietary analysis, with the potential to determine estimates of diet composition [23], [40], [41]. Thirdly, a number of recent studies have highlighted the potential impact that High-Throughput Sequencing (HTS) may have on dietary studies. HTS has been proposed as a cost-effective alternative in assessing and quantifying species' diet [14], [16], [21], and using indexed primers enables a large number of samples to be processed in parallel [14], [42], [43]. As yet, however, no study has validated the use of HTS in providing quantitative estimates similar to those obtained via qPCR.

This study sets out to determine the composition of Little Penguin faecal samples by comparing cloning, qPCR and HTS approaches. The primary purpose of this study was to develop an effective long-term strategy for the continual monitoring of diet in the penguin population. However, it is envisaged that the approach and recommendations advocated here will assist in experimental design for DNA-based faecal monitoring across a wide diversity of species.

## Materials and Methods

The handling of penguins and the collection of faecal samples was conducted by experienced handlers under a strict set of animal ethics guideline approved by the Murdoch University Animal Ethics Committee (permit no. W2002/06).

### Sample collection & storage

A total of 47 penguin faecal samples were collected, for cloning analysis, over the period from August 2008 until September 2009 and a further 52 samples, for HTS and qPCR analyses over the period from October to December 2010. All samples were collected from free-living penguins inhabiting the study area (Fig. 1). Samples were collected opportunistically from adults and chicks by checking artificial nest boxes or by intercepting penguins returning from the ocean to their nests. Adult penguins were placed in plastic-lined containers for a maximum of 15 minutes. Chicks were placed in a smaller container with a hot water bottle for a maximum of 15 minutes before being returned to their nest boxes. Upon collection the faecal samples were placed in a labelled vial and then stored at −20°C within 12 hours. All handling and sampling was carried out under Murdoch University Animal Ethics Committee permit W2002/06.

### Sample preparation & DNA extraction

The penguin samples were extracted in batches with the appropriate extraction controls. Samples were weighed and collected into 2mL tubes, with between 26–330mg of sample being used in each extraction depending on the condition of the faecal material. Extractions were performed using QIAamp DNA Stool Mini Kit (QIAGEN) as per manufacturer's instructions. DNA was eluted in 100 µL of AE buffer and dilutions of 1∶10 and 1∶50 were made using Milli-Q UV Pure H_2_O for subsequent PCR reactions. DNA extracts were stored at −20°C until further analyses were performed.

### Sample screening & initial quantification

Each faecal extract was screened using qPCR with 16S1F/2R primers in order to assess the DNA quality, quantity and to detect any possible PCR inhibition [44] (Table 1). Each extract was amplified at neat, 1∶10 and 1∶50 dilutions using the ABI Step One Real Time PCR machine. Each reaction was made up to 25 µL, containing 12.5 µL Power Sybr master mix (Applied Biosystems), 0.4 µM of each primer, 8.5 µL H_2_O and 2 µL DNA. Reaction conditions were as follows: initial heat denaturation at 95°C for 5mins, followed by 40 cycles of 95°C for 30s; 54°C for 30s; 72°C for 45s followed by final extension at 72°C for 10mins and a 1°C melt curve to assist in the identification of primer dimer and non-specific amplification.

**Table 1:** List of primer pairs used in this study.

Target species	Primer name	Sequence (5′-3′)	Product Size (bp)	Annealing temp. (°C)	Ref.
*Engraulis australis*	AN1F*	CCTAAATACCCGCAGCCTTAT	101	60	This study
(Australian Anchovy)	AN2R*	CAACTCTCGGCTTAAGGGTTT			
*Spratelloides robustus*	BS2F*	GCGGCTACTGCCCTAACTATCGC	109	60	This study
(Blue Sprat)	BS2R*	CTGAGCTCCAGGCCGAAGGC			
*Sardinops sagax*	PIL1F*	CCTAACTGGAGCCCCAAAC	117	60	This study
(Australian Pilchard)	PIL1R*	GCTGTGGCTCTGGGTTTTAG			
*Hyperlophus vittatus*	SS2F*	GGCCTCAAACAACATGACAGT	91	60	This study
(Sandy Sprat)	SS2R*	TAGGGTGGCCCTAATCCACT			
All prey	16S1F-degenerate^¶^	GACGAKAAGACCCTA	180–270	54	[44]
	16S2R-degenerate^¶^	CGCTGTTATCCCTADRGTAACT			

Primers listed include species specific pairs (*) used in the targeted four fish qPCR assays and the universal pairs (^¶^) used in cloning and High Throughput Sequencing approaches. Note the 16S1F/16S2R primers had 5′ fusion and MID tags [46] if they were to be sequenced on the GS-Junior.

### Cloning of amplified DNA

PCR products were cloned into pGEM®-T vectors (Promega) following the manufacturer's protocol and a maximum of 10 positive clones were selected per sample and amplified using the M13F/M13R primer set. Each 25 µL reaction contained 1X PCR buffer, 2mM MgCl_2_, 0.4mg/mL BSA, 0.25mM each dNTP, 0.6 µL *SYBR* Green (Invitrogen), 0.4 µM of each primer, 0.25 µL *Taq* polymerase and 2.0 µL of template DNA. The cycling conditions were as follows: initial denaturation at 94°C for 5mins, followed by 35 cycles at 94°C for 15s; 55°C for 30s; 72°C for 30s. Amplicons were purified using an ACROPrep 10K 96 well plate (Pall) under a 25mmHg vacuum and screened via gel electrophoresis. Amplicons of the correct size were sequenced by Macrogen (Korea) using BigDye sequencing chemistry (Applied Biosystems) and analysed using Geneious v5.4.6 [45].

### HTS library preparation

Prior to amplicon sequencing on the GS-Junior (454 Life Sciences), the 16S1F and 16S2R-degenerate primers were modified into fusion primers consisting of a GS FLX Titanium Primer A or B on the 5′ end followed by one of 25 different 6bp Multiplex Identifier (MID) tags (allowing the simultaneous processing of 25 different PCR products) and then the template specific primer at the 3′ end [46].

Extracts that successfully yielded DNA, as determined by the initial screening via qPCR, were assigned a unique tagged primer set. Fusion tagged PCR was carried out in 25 µL reactions containing 1X PCR Gold Buffer, 2.5mM MgCl_2_, 0.4mg/mL BSA, 0.25mM each dNTP, 0.4 µM of each primer, 0.25 µL AmpliTaq Gold (Applied Biosystems) and 2 µL DNA. The cycling conditions were as follows: initial heat denaturation at 95°C for 5mins, followed by 40 cycles of 95°C for 30s; 54°C for 30s; 72°C for 45s followed by final extension at 72°C for 10mins. Amplicons were always generated in duplicate and pooled together to minimise the effects of PCR stochasticity. The resultant pooled amplicons were purified using Agencourt AMPure XP PCR Purification Kit (Beckman Coulter Genomics, NSW, Aus), and eluted in 40 µL H_2_O. Purified amplicons were electrophoresed on 2% agarose gel and amplicons were pooled in approximately equimolar ratios based on band intensity.

### GS-Junior set-up and sequencing

To achieve the desired bead:template ratio, pooled amplicons were quantified using a synthetic 200bp oligonucleotide standard (of known molarity) with the Roche A and B primers engineered at either end. Quantitative PCR on a dilution series of both the standard and the pooled library, each run in duplicate, has enabled us to reproducibly normalise bead:template ratios. All procedures involved in the set up of the sequencing run (emulsion PCR and bead recovery), including the sequencing run itself, were carried out according to the Roche GS Junior protocols for amplicon sequencing (http://www.454.com).

### 2.7 Four fish qPCR assay

Based on previous diet studies [24]–[27], [31] and the DNA sequence data it was apparent that *Engraulis australis* (Australian Anchovy), *Spratelloides robustus* (Blue Sprat), *Sardinops sagax* (Australian Pilchard) and *Hyperlophus vittatus* (Sandy Sprat) formed a major part of the Little Penguins' diet. Therefore, in order to quantitatively assess the abundance of each of these species within each faecal sample and also to compare the quantitative nature of HTS using degenerate primers to that of qPCR, species-specific primer pairs (Table 1) were designed for each of the four fish species using Geneious v5.4 [45]. Primer sets for the four fish were designed using regions within the mitochondrial genes encoding for 16S rRNA based on sequence data obtained from local fish. Each primer pair was tested for efficiency and sensitivity on their target fish species. Importantly, the primer pairs were selected only if they did not cross-react with each other or other species detected in the area [27], [47]. Once primer pairs were optimised, qPCR of faecal samples that successfully yielded DNA were performed in 25 µL reactions containing 1X PCR Gold Buffer, 2.5mM MgCl_2_, 0.4mg/mL BSA, 0.25mM each dNTP, 0.4 µM of each primer, 0.25 µL AmpliTaq Gold and 0.6 µL SybrGreen (Invitrogen cat no S7563, 1∶2000 dilution). Cycling conditions were as follows; initial denaturation at 95°C for 10min, followed by 40 cycles of 95°C for 15sec; 60°C for 45 sec.

### Data analysis

FASTA (.fna) and Quality (.qual) sequence files obtained from the GS FLX Junior sequencing runs were processed using the following programs; BARTAB [48] de-convoluted the reads into sample batches using a map file containing sample and primer-MID tag information, cross_match [49] masked the primer and MID-tag sequences contained in the map file, trimseq [50] trimmed the masked primer and MID-tag sequences, and finally each sample of batched reads was then searched using BLASTN [51] without a low complexity sequence filter against the NCBI GenBank nucleotide database [52]. This was automated in the Internet-based bioinformatics workflow environment, YABI [https://ccg.murdoch.edu.au/yabi/]. The BLAST results that were obtained using YABI were imported into MEtaGenome Analyzer (MEGAN) where they were taxonomically assigned using the LCA-assignment algorithm (parameters included: min. bit score  = 65.0, top percentage  = 10%, min. support = 1) [17]. Where MEGAN was unable to resolve the taxonomy of a sequence (due to multiple species' sequences matching the query sequence), taxonomies were assigned using a combination of FishBase [http://fishbase.org] and Atlas of Living Australia [http://www.ala.org] to determine the most likely species based on their geographic distribution. Where more than one species returned by GenBank occurred around the Perth coastal area the query sequence was assigned to a higher taxonomic level.

Upon successful classification of all sequences obtained via HTS the percentage contribution of each prey item identified within each faecal sample was calculated, in addition to the overall contribution of each prey item across all faecal samples. In the case of the cloning data, a presence/absence method was used to determine the abundance of prey items within faecal samples.

In order to calculate the percentage contribution of each of the four major fish species within each faecal sample during the Oct ‘10-Dec ’10 sampling period, the C_T_ (Cycle threshold) values obtained for the four target species via qPCR (at the same dilution if deemed free of inhibition) were compared and converted into a percentage relative to each other. These individual percentages were then used to calculate the overall proportion of each of the four fish species across all faecal samples. Due to the stochasticity associated with low copy number DNA and primer dimer accumulation above C_T_ values of 34, all C_T_ values recorded above this level were attributed a C_T_ value of 34. This approach enables the target amplicon's presence to be acknowledged, whilst still allowing for it to be expressed proportionally to the other fish species within that sample.

To enable comparison of the qPCR and HTS datasets, the proportions of each of the four major fish species within each faecal sample as determined via HTS were considered to the exclusion of all other prey species detected. Using these data in conjunction with that obtained via qPCR, the Pearson product-moment correlation coefficient (Pearson's *r*) was calculated to determine the degree of correlation between the datasets. Additionally, individual paired sample *t*-tests for each major fish species were used to determine if there was a significant difference between the data obtained via both methods for any of the four major fish species. Samples that recorded C_T_ values >34 were excluded from statistical analyses, due to the stochasticity of qPCR above this threshold. All statistical analyses were carried out using the program R.

## Results and Discussion

### Overview and comparisons of Cloning and HTS approaches

**Figure 2:**
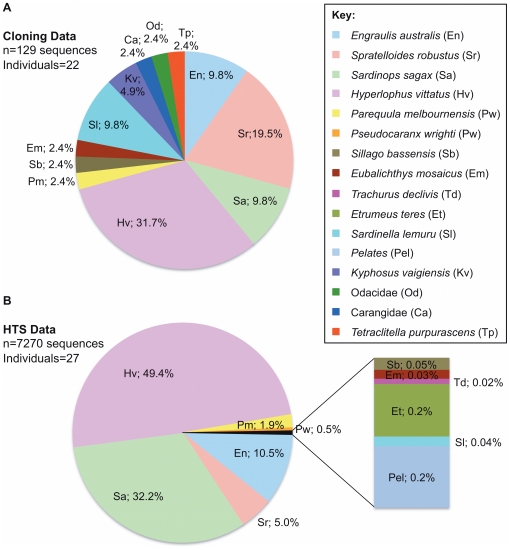
Percentage contribution of identified prey items in the faecal DNA of *E. minor*. (**A**) Graph showing fish identifications based on 16S rRNA sequence data obtained via cloning using universal primer set 16SF1/16S2R. Faecal samples (n = 22) for this study were collected during the Sep ‘08/Aug ’09 period. (**B**) Penguin faecal samples collected during Oct ‘10-Dec ’10 period (n = 27) that were audited using HTS methods. The 16SF1/16S2R set were MID-tagged and a total of 7270 sequences were assigned to prey items.

Using the cloning approach, a total of nine fish species were identified from 129 sequences, in 22 of the 47 samples (47%) collected during the Aug ‘08-Sep ’09 sampling period. Samples deemed to have failed either yielded no amplifiable DNA, were severely compromised by inhibitors, or had target copy numbers (as determined by qPCR C_T_ values >35.0) that were considered too low to be reliable. The dominant prey species detected within these samples was *H. vittatus*, present in 32% of samples, followed by *S. robustus*, found in 20% of samples, with *S. sagax*, *E. australis* and *Sardinella lemuru* (Scaly Mackerel) each found in 9.8% of samples (Fig. 2A). A number of other minor prey items were also identified, however they were found to represent a small proportion of sequences (Fig. 2A).

Of the 52 samples collected during the Oct ‘10-Dec ’10 sampling period, only 27 samples (52%) were deemed to have yielded DNA of sufficient quality free of inhibition (determined by qPCR) that they could advance to HTS analysis. The two independent GS-Junior runs generated a total of 7810 DNA sequences. Of these sequences ∼93% were unambiguously attributed to eleven fish species and <0.1% were identified as belonging to the genus *Pelates* (Striped Grunters). There were low levels of human contamination and penguin DNA (∼3%) and unassigned/uninformative sequences accounted for ∼3.6% of sequences. There was notable variation in the number of sequences generated for each faecal sample (range = 35–1055), and this is likely due to inaccurate blending of amplicons (see Materials & Methods). However, an average of ∼300 reads per sample is more than sufficient coverage for dietary audits, especially when compared to the average number of sequences often generated per sample using bacterial cloning [53], [54]. HTS of the Oct ‘10-Dec ’10 samples revealed that, of the prey items identified, *H. vittatus*, *S. sagax*, *E. australis* and *S. robustus* were the major species present within the faecal material, each contributing 49%, 32%, 11% and 5% respectively (Fig. 2B). The remaining fish identified were minor contributors to the overall composition of the samples (ranging from 0.02% to 1.9%) (Fig. 2B) and only in one sample did any of these fish constitute a significant proportion of the prey detected, that of PEN_42, where *Parequula melbournensis* (Silverbelly) contributed 48% to the sample composition for this individual (Table S1).

It is clear from the bacterial cloning and HTS data that there were four dominant fish species detected within the samples at this study site, those being *H. vittatus*, *S. sagax*, *E. australis* and *S. robustus* (Fig. 2). The occurrence of other minor contributing prey items within the samples is consistent with previous findings and reflects the opportunistic feeding behaviour of the Little Penguins [24], [27]. A direct comparison of cloning and HTS is somewhat hampered by the fact that different faecal samples from different time periods were used for each method. However, it is clear that a number of important conclusions can be drawn from both datasets. Both methods provide a clear picture of the major prey species that are present within the collective faecal samples. Where they differ is in the relative contribution of each of these individual species (Fig. 2), however this could be a result of temporal effects as it is well documented that the diet of Little Penguins varies throughout the year [27].

Cloning of universally amplified PCR products using bacteria, followed by DNA purification and Sanger sequencing is both expensive and time consuming. An additional issue, not entirely observed in this study, is that large numbers of clones are required in order to detect rare species [5], [53], with the associated time and expense being inefficient for long-term monitoring of species' diet. For this reason, our Little Penguin monitoring program made the transition to HTS for the 2010 samples. Newly developed HTS platforms, especially small-scale systems such as the GS-Junior or IonTorrent, enable a quick, efficient and relatively inexpensive way to deep-sequence PCR amplicons generated from faecal DNA extracts [14], [16], [21]. Moreover, the use of MID-tagged primers makes it possible to run numerous samples in parallel, enabling not only an overview of the diet composition across a population, but also at the individual level [14], [42]. HTS can provide a wealth of information; greatly increasing the number of DNA sequences returned (129 sequences vs 7810 sequences) for a fraction of the labour and associated costs. Concomitant with the increases in sequencing depth is the prospect that HTS data might now provide better quantitative measures of the DNA targets within faecal material, much like estimates obtained using qPCR [23], [44].

### Overview of qPCR approach

**Figure 3:**
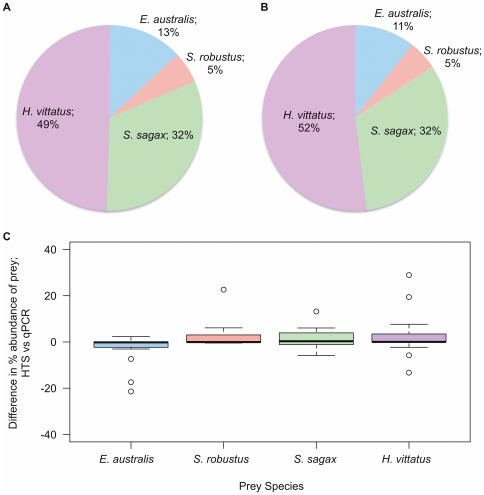
Comparison of HTS and qPCR methods determining the proportion of four major fish species. Graphs indicate the relative percentage composition of *H. vittatus*, *S. sagax*, *E. australis* and *S. robustus* within faecal samples of *E. minor* on Penguin Island, as determined by (**A**) qPCR and (**B**) HTS of samples collected during the period of Oct ‘10-Dec ’10. (**C**) Box plot showing the difference between the results obtained by HTS and qPCR for each of the four major fish species found in the diet of *E. minor.* Samples whose C_T_ values were >34 have been excluded from the dataset (see Materials and Methods).

In order to compare the quantitative nature of HTS to that of qPCR, a species-specific four fish qPCR assay was designed to estimate the relative abundance of each of the four major prey species determined within the collective samples (Fig. 2, Table 1). Careful development of each of the four primer pairs was critical to data fidelity [19], [55], as was ensuring that the DNA extracts' C_T_ values behaved as desired when diluted (i.e. they were free from inhibition). From this four fish assay it was clear that *H. vittatus* and *S. sagax* were major constituents of the faecal samples; 49% and 32% respectively, with both *E. australis* and *S. robustus* each contributing 13% and 5% to the overall composition (Fig. 3A). The ANF1/ANR2 assay encountered some primer dimer issues at low template copy numbers, however the melt curves enabled differentiation of product and dimer. Although not wholly representative of the *total* amount of prey DNA within samples, the qPCR assays gave a good indication of the abundance of each of the four major fish species relative to each other.

### Comparison of HTS & qPCR approaches

**Figure 4:**
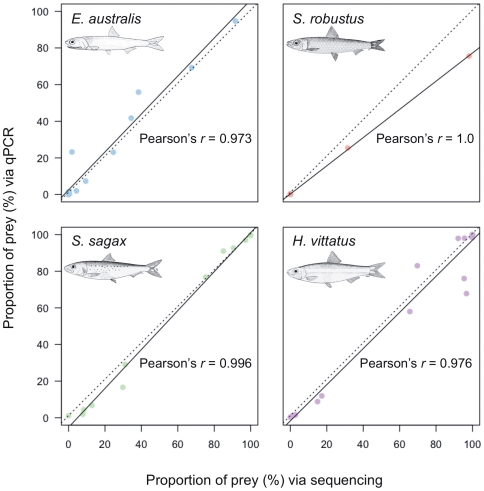
Correlation between four-fish data obtained via HTS and qPCR. Scatterplots include the percentage contributions obtained for each individual penguin via HTS and qPCR for each of the four major fish species detected within faecal samples. Solid line represents the line of best fit for individual species (Pearson's *r* values are shown), whilst the dotted line represents the overall correlation between both datasets with the data obtained for all fish species across all samples combined. Samples whose C_T_ values were >34 have been excluded from the dataset (see Materials and Methods). Fish images used in this figure can be reproduced freely for non-commercial purposes and are sourced from [59].

It is important to actively compare and contrast both HTS and qPCR approaches to enable an informed decision of the most suitable method to be used for genetic faecal screening. To allow a comparison between both approaches, the HTS data had to be transformed to focus on the same four fish species as the qPCR assay; *H. vittatus*, *S. sagax*, *E. australis* and *S. robustus.* The proportion of these species to the exclusion of the other species present was determined to be 52%, 32%, 11% and 5% respectively (Fig. 3B transformed from fig 2B data). It is clear that there is a striking degree of similarity between the proportions identified for the four fish species determined by qPCR and HTS (Fig. 3C). In order to investigate this further, the absolute differences between the results obtained individually by both methods were calculated. In the case of each fish species the overall difference in percentage abundance between the two techniques was negligible (*H. vittatus* - Median = 0.02, n = 19; *S. sagax* - Median = 0.31 n = 13; *E. australis* - Median =  −0.18, n = 15; *S. robustus* - Median =  −0.05, n = 7) (Fig. 3C). These initial results demonstrate a high degree of similarity between individual measures obtained by both methods. Furthermore, Pearson's *r* calculations revealed strong correlations between both methods for all four fish species (*H. vittatus* – Pearson's *r* = 0.976, n = 19; *S. sagax* - Pearson's *r* = 0.996, n = 13; *E. australis* - Pearson's *r* = 0.973, n = 15; *S. robustus* - Pearson's *r* = 1.0, n = 7) (Fig. 4), whilst individual paired *t*-tests revealed no significant difference between the values obtained by either method for any of the major prey species (*H. vittatus* – *p* = 0.215, n = 19; *S. sagax* - *p* = 0.226, n = 13; *E. australis* - *p* = 0.100, n = 15; *S. robustus* - *p* = 0.266, n = 7).

Although no statistical difference was detected in species composition in the combined analysis, it was apparent that there are slight differences between the datasets at the individual level (Table S2). There could be a number of reasons for such differences. Firstly, differential degradation of prey tissue DNA could account for some of the variance between datasets [23], [39]. In this study the amplicon sizes produced by the primer sets in qPCR were shorter than those for HTS (see Table 1), and so in some instances length biases may be present, especially in instances where there is differential degradation of prey tissue DNA in the gastrointestinal tract [41]. Indeed, it would appear that in this study *E. australis* was slightly over-represented in qPCR relative to HTS, whilst *H. vittatus* was marginally under-represented in qPCR relative to HTS (Table S2). A second potential cause could be the fact that the targeted qPCR assay is more efficient than the universal 16S primers used in HTS, therefore enabling the detection of the four prey species' DNA at lower template amounts. This is best illustrated when considering the presence/absence data, where HTS vs qPCR detection rates are compared: 70.4% vs 88.9% (*H. vittatus)*, 48.2% vs 81.5% (*S. sagax)*, 40.7% vs 74.1%(*E. australis)* and 14.8% vs 40.7% (*S. robustus).* In all cases where a species was detected in qPCR but not in HTS the C_T_ values were either >34 or the relative abundance of that species was below 1.5% (Table S2). Taken together, these data do suggest that the shorter, targeted qPCR assays were, across all four fish species, more sensitive to low template amounts. However, the higher qPCR detection success did not drastically affect the overall estimates of both methods, due to the low abundance of prey species in these instances. This also highlights a very important advantage of species-specific qPCR over HTS, in that it can detect species at very low DNA abundances, whereas the nature of universal primers, such as those used in HTS, renders them less specific and less likely to efficiently amplify low copy number targets in the presence of abundant targets.

Whilst it is clear that there are slight differences between both methods, which are attributable to a variety of factors, it is also clear that in this case no single factor seemed to have a detrimental effect on the overall estimates of prey items within the collective faecal samples. It appears, however, that the difficulty arises when the penguins are considered on an individual basis. If, for instance, HTS were solely used in this study then it is quite clear that a good idea of the overall breadth of species could be ascertained. However, in some cases the use of universal primers may result in the non-detection of certain dietary constituents, if present in low abundance. On the other hand, with the use of the targeted qPCR approach a possibly more accurate estimate of the relative contribution of the major fish species' DNA could be determined across the population and individually, provided an *a priori* knowledge of diet is known. However, the contribution of the other minor constituents is overlooked. It would appear that the effect of this is largely minimal, unless, as was the case with sample PEN_42, one of the ‘minor contributors’ accounts for a large proportion, or all, of any given sample.

### Recommendation for future experimental design

The uptake of genetic techniques to analyse faecal material has provided important insights into animal diet. It is clear that the use of qPCR and the advent of affordable HTS technologies are proving to be a welcome addition to this field of research. Both of these techniques have the potential to eclipse the more traditional molecular methodology of bacterial cloning and/or direct sequencing, which is costly, laborious and time-consuming. In light of the results of this study, it is fair to assume that qPCR and HTS represent the best approach currently available.

A key component of experimental design in this study was the methodical preparation and selection of samples for DNA extraction prior to qPCR or HTS. The extraction of DNA from faecal samples and the screening of samples for copy number and inhibition is a major bottleneck in the lab. However, the importance of this screening process cannot be under-stated, particularly when the samples being dealt with are complex, heterogeneous substrates containing severely degraded DNA in low copy numbers [56], [57]. The initial qPCR screening strategy implemented in this study allowed the identification of suitable samples and DNA extract dilutions that contained the maximum concentration of amplifiable DNA and yet were inhibition free. There is no substitute for prior screening of samples; the congruence of qPCR and HTS in this study can be attributed largely to the fact that there is confidence in the amplifiability of the DNA extract dilution on which HTS and qPCR was conducted.

The ultimate choice of which method to opt for should be considered on a case-by-case basis, although the use of both methods in tandem would be the preferred option. If, for instance, an *a priori* knowledge of the species' diet in question were lacking then it would be more appropriate to use HTS with universal primer sets, thus giving an overview of the animal's diet. With this broad view of the animal's diet it can then be decided whether to pursue the use of targeted primers via the qPCR approach. If the number of prey species within the diet is of limited complexity qPCR may be preferable. Although not implemented here, in theory the quantitativeness of HTS using universal primers could be improved by using multiple universal primer sets in parallel [7], [21].

If the goal of any dietary study is the long-term monitoring of diet, then it would be advisable to use HTS to determine the overall composition of the diet, and if possible a subsequent targeted qPCR approach to examine major prey items, to ensure that the diet remains consistent throughout the period of study. Ideally it would be beneficial to consider the use of both techniques in parallel to safeguard against erroneous results, as the removal of major contributors to the diet can have profound impacts on prey quantification. This is highlighted by the example of PEN_42 where *P. melbournensis* formed a major part of that individual penguin's faecal sample (Table S1). Therefore, in this case, the four fish qPCR assay is a poor representation of prey abundance.

Irrespective of the chosen method, primer design is crucial to the sensitivity of PCR, and careful consideration should be given to the design and testing of primers [19]. In the case of universal primers used in HTS, it is imperative that they are designed to allow taxonomic discrimination of amplicons, and yet also amplify a small enough region to circumvent issues of DNA degradation within faeces [19]. One additional issue is the fact that the coverage of certain animal groups in certain databases is not complete which will always make taxonomic assignments difficult [5], [14]. The study of bats is a case in point; in this instance the use of qPCR assays would not be able to account for the hundreds of insects species in bat guanos, however qPCR could still be used to validate the relative portion of a few target species [5], [14].

The validation of the quantitative nature of HTS, as compared to qPCR, to detect the DNA in faecal material, bodes well for future dietary studies. However, it is acknowledged that the results obtained via DNA-based faecal analysis are not always directly correlated with the biomass of prey consumed [55] – a recent study referred to them as semi-quantitative at best [23]. Much work is yet to be done to enable accurate reconstructions of the physical diet as estimates are currently confounded by a range of factors including; differential digestion rates of prey between species; DNA per unit biomass variability between tissues and the developmental stage of the prey species to name but a few issues [23], [42], [58]. It is also questionable whether digestion/faecal studies of captive birds will accurately recreate what is happening in the wild. Despite the many caveats regarding actual dietary intake, the accurate quantification of prey DNA actually contained in faecal matter represents an important developmental step.

### Conclusion

Characterising the DNA preserved in faecal material is a powerful way to study both animal diet and also provide broader insights into ecosystem composition and health. In light of recent advances in DNA sequencing it was unclear which genetic auditing method(s) should be adopted for a multi-year monitoring program of Little Penguins. The results of qPCR and HTS approaches tested in this study demonstrate that the two methods are capable of generating high-fidelity datasets with no statistical difference between them. In the case of penguin diet, the use of both methods in parallel proved particularly useful with species-specific qPCR assays having better sensitivity, whilst HTS is able to detect species not targeted by qPCR. It is anticipated that the data and approaches presented here will be of benefit to other researchers intending to implement dietary monitoring programs and will assist in improving the accuracy of environmental audits based on faecal material.

## Supporting Information

Table S1 **Percentage contribution of prey items detected by HTS for each faecal sample.** The percentage contribution of detected prey items within each individual faecal sample, as determined by HTS of samples collected during the period of Oct ‘10-Dec ’10, using 16SF1/16S2R universal primers.
(XLS)

Table S2 **Percentage contribution of four major fish species determined by HTS and qPCR methods.** The percentage composition of *H. vittatus*, *S. sagax*, *E. australis* and *S. robustus* within individual faecal samples of *E. minor* on Penguin Island, as determined by HTS and qPCR of samples collected during the period of Oct ‘10-Dec ’10.
(XLS)
